# A Case of the Monocle Sign in Facial Nerve Palsy Caused by External Auditory Canal Cancer on 18F-fluorodeoxyglucose-positron Emission Tomography/Computed Tomography

**DOI:** 10.31662/jmaj.2024-0303

**Published:** 2025-03-14

**Authors:** Reiko Yagi, Ken Yamagiwa, Tomoyuki Fujioka, Junichi Tsuchiya, Tomoaki Asamori, Takeshi Tsutsumi, Ukihide Tateishi

**Affiliations:** 1 Department of Diagnostic Radiology, Institute of Science Tokyo, Tokyo, Japan; 2 Department of Head and Neck Surgery, Institute of Science Tokyo, Tokyo, Japan; 3 Department of Otolaryngology, Institute of Science Tokyo, Tokyo, Japan

**Keywords:** external auditory canal cancer, facial nerve paralysis, PET/CT

A 47-year-old woman with right external auditory canal cancer presented with an inability to close her left eyelid. Contrast-enhanced computed tomography (CT) identified a tumor extending from the right external auditory canal to the petrous bone, infiltrating the right facial nerve (Figure 1). ^18^F-fluorodeoxyglucose (^18^F-FDG)-positron emission tomography (PET)/CT revealed intense ^18^F-FDG uptake at the site. Notably, the left orbicularis oculi muscle (OOM) showed ^18^F-FDG uptake (maximum standardized uptake value: 3.08), whereas the right OOM showed no abnormal uptake (Figure 2).

**Figure 1.**
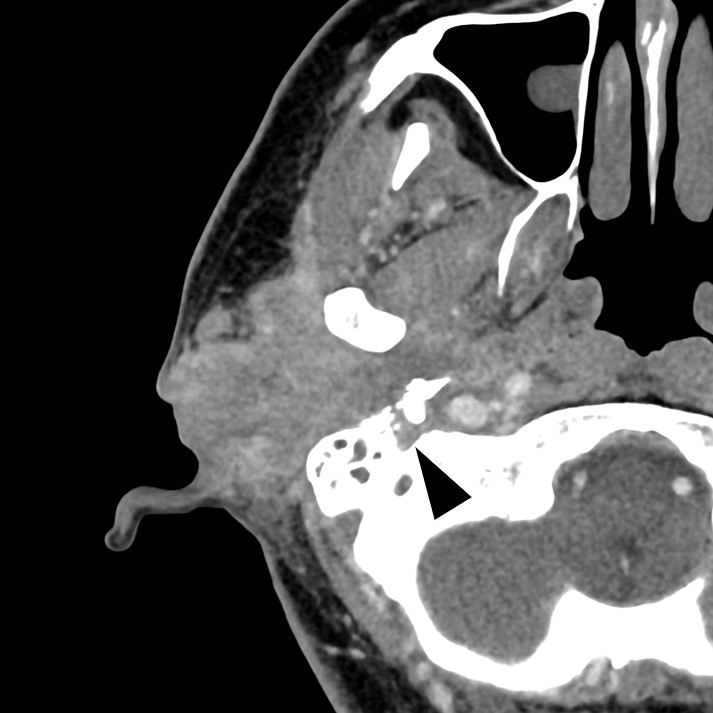
Contrast-enhanced computed tomography shows a contrast-enhanced tumor extending from the right external auditory canal to the piriformis and invading the right facial nerve. The arrowhead indicates the tumor extending into the stylomastoid foramen.

**Figure 2.**
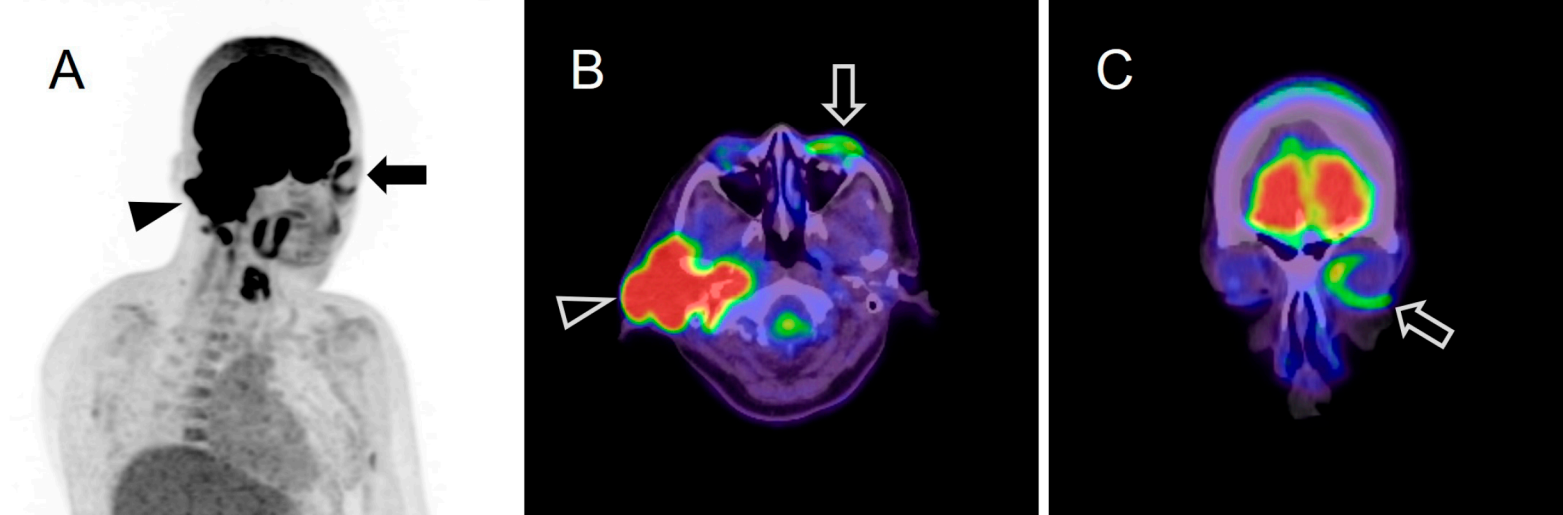
Positron emission tomography/computed tomography showing intense ^18^F-FDG uptake associated with right external auditory canal cancer (A, B: arrow heads) and increased ^18^F-FDG uptake (maximum standardized uptake value: 3.08) in the left orbicularis oculi muscle on the unaffected side (A, B, C: arrows). ^18^F-FDG: ^18^F-fluorodeoxyglucose.

Asymmetrical uptake by the OOM, referred to as the “monocle sign,” indicates contralateral peripheral facial nerve palsy (FNP) ^(1)^, which is believed to result from the overactivity of the nonparetic OOM. To the best of our knowledge, this is the first reported case of the monocle sign in a patient with external auditory canal cancer and FNP ^(1), (2)^.

Radiologists should recognize the monocle sign to prevent misdiagnosing unilateral ocular uptake under other conditions.

## Article Information

### Conflicts of Interest

None

### Acknowledgement

We used GPT-4o (https://chatgpt.com/) for Japanese-English translation and English proofreading. The authors read, revised, and proofread the generated text.

### Author Contributions

Reiko Yagi wrote the first draft of the manuscript, and Tomoyuki Fujioka, Ken Yamagiwa, Junichi Tsuchiya, Tomoaki Asamori, and Takeshi Tsutsumi revised the manuscript. Tomoaki Asamori and Takeshi Tsutsumi contributed to patient care. Ukihide Tateishi supervised all the procedures.

### Informed Consent

We have obtained informed consent for this case report.

### Approval by Institutional Review Board (IRB)

Not applicable.
